# Overproduction of a Model Sec- and Tat-Dependent Secretory Protein Elicits Different Cellular Responses in *Streptomyces lividans*

**DOI:** 10.1371/journal.pone.0133645

**Published:** 2015-07-22

**Authors:** Sonia Gullón, Silvia Marín, Rafael P. Mellado

**Affiliations:** Departamento de Biotecnología Microbiana, Centro Nacional de Biotecnología (CNB-CSIC), c/Darwin 3, 28049, Madrid, Spain; Centre National de la Recherche Scientifique, Aix-Marseille Université, FRANCE

## Abstract

*Streptomyces lividans* is considered an efficient host for the secretory production of homologous and heterologous proteins. To identify possible bottlenecks in the protein production process, a comparative transcriptomic approach was adopted to study cellular responses during the overproduction of a Sec-dependent model protein (alpha-amylase) and a Tat-dependent model protein (agarase) in *Streptomyces lividans*. The overproduction of the model secretory proteins via the Sec or the Tat route in *S*. *lividans* does elicit a different major cell response in the bacterium. The stringent response is a bacterial response to nutrients’ depletion, which naturally occurs at late times of the bacterial cell growth. While the induction of the stringent response at the exponential phase of growth may limit overall productivity in the case of the Tat route, the induction of that response does not take place in the case of the Sec route, which comparatively is an advantage in secretory protein production processes. Hence, this study identifies a potential major drawback in the secretory protein production process depending on the secretory route, and provides clues to improving *S*. *lividans* as a protein production host.

## Introduction

The overproduction of homologous and heterologous proteins for pharmacological and industrial application requires the use of different prokaryotic and eukaryotic expression systems. The use of prokaryotic expression systems reduces the cost of the process owing to the inexpensive culture media and it has moreover been proven to obtain high expression levels of the secreted proteins [1]. Gram-positive bacteria are naturally producers of extracellular proteins that are secreted to the medium, thus simplifying the complex purification procedures inherent to intracellular protein accumulation. Streptomycetes are Gram-positive GRAS (generally recognized as safe) soil bacteria, providing a huge secretion capacity of hydrolytic enzymes together with antibiotics and signalling molecules [2] to adapt to their natural environment largely formed of insoluble polymers. *Streptomyces lividans*, in particular, has a relatively inefficient restriction-modification system and low endogenous protease activity when compared to many other streptomycetes, hence it has been used for the secretory production of heterologous and homologous proteins [3], achieving the secretion of proteins which otherwise could not be produced in the Gram-negative bacteria *E*. *coli* or in the Gram-positive bacteria *B*. *subtilis* [4, 5, 6]. However, in some cases, low yields were obtained [3]. To improve protein production, transcriptomic studies of the cells overproducing secretory proteins in *S*. *lividans* have been performed in order to identify the potential bottlenecks that may limit the yield of the secreted protein, thus enabling the optimization of protein production. *Streptomyces* make use of two main pathways to target secretory proteins to the cytoplasmic membrane: the major Sec pathway that secretes proteins in a yet unfolded conformation, and the Tat pathway that secretes proteins in a folded conformation. The Tat pathway is a minor pathway in *Streptomyces*, as in other bacteria, although the number of potential Tat substrates is greater [7].

In this work a comparative transcriptomic approach was conducted to study cellular responses when a Sec-dependent protein (alpha-amylase) [8] and a Tat-dependent protein (agarase) [9] were overproduced in *S*. *lividans*.

The overproduction of secreted proteins using the Sec and Tat route in *S*. *lividans* seems to elicit different cell responses in bacteria. The overproduction of agarase protein leads mainly to a downregulation of ribosomal gene expression, which, among others, has been reported to form part of a stringent response in *Streptomyces*. However, the overproduction of alpha-amylase protein results in an increased level of ribosomal gene expression and that of other genes associated with active cell growth. Thus, the overproduction of proteins using the Tat system causes a potential earlier depletion of precursors that may lead to cellular death, while engineering the secretion of extracellular proteins via the Sec route may ensure a more efficient production of secretory proteins, apparently causing no metabolic damage to the cell.

## Results

### Expression of genes modulated by alpha-amylase and agarase overproduction

To study cellular response when overproducing a Sec-dependent protein (alpha-amylase, AmlB) or a Tat-dependent protein (agarase, DagA), the *S*. *lividans* alpha-amylase gene (*amlB*) or the *Streptomyces coelicolor* agarase gene (*dagA*) were propagated in multicopy plasmids in *S*. *lividans* TK21 harbouring *amlB* (pAM11) [8] or *dagA* (pAGA5) [10], under the control of their own promoters, respectively.

**Fig 1:**
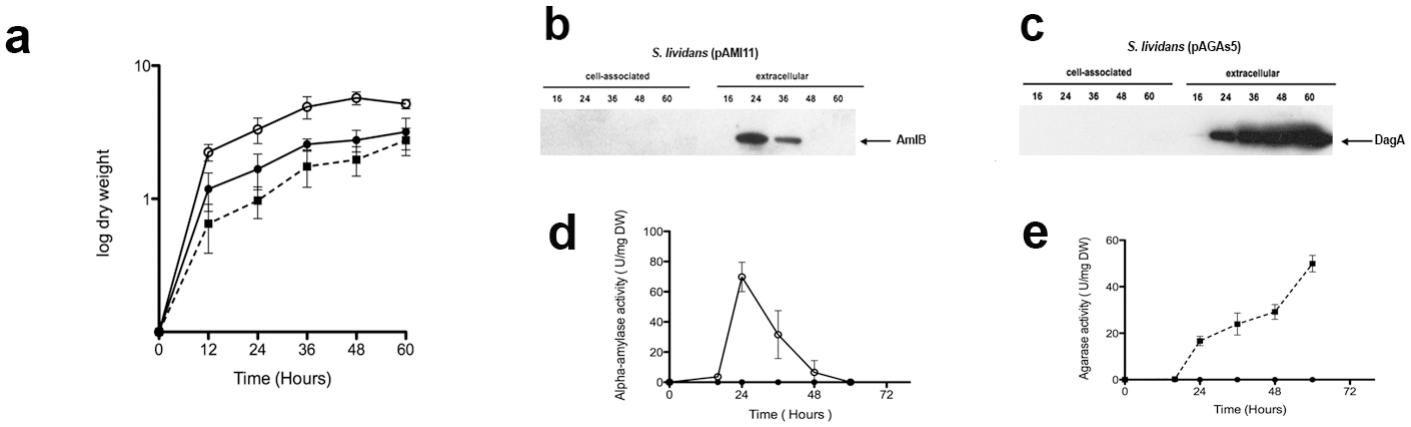
Growth curves, western blot and extracellular activity of the overproducer strains. (a)Time-course of the *S*. *lividans* TK21 (pIJ486) (black circles), *S*. *lividans* TK21 (pAMI11) (white circles) and *S*. *lividans* TK21 (pAGAs5) (black squares) bacterial cell cultures growing in NMMP medium. The values are the mean of at least three biological replicates. Bars show standard error. Cell-associated and extracellular amylase present in *S*. *lividans* TK21 (pAMI11) (b), agarase present in *S*. *lividans* TK21 (pAGAs5) (c) at different times of growth were analysed by Western blotting with antibodies raised against AmlB and DagA. The amount of protein loaded onto the gels was corrected by the dry cell weight of the bacterial cultures. An arrow indicates the relative mobility of the proteins. (d) Alpha-amylase activity present in *S*. *lividans* TK21 (pAMI11) (white circles) and in *S*. *lividans* TK21 (pIJ486) (black circles) was determined. (e) Agarase activity present in *S*. *lividans* TK21 (pAGAs5) (black squares) and in *S*. *lividans* TK21 (pIJ486) (black circles) was determined. The specific extracellular activities were expressed as units per mg of dry weight. The data are the average of at least three independent determinations.

The agarase overproducer strain revealed a greater tendency to aggregate in clumps when grown in liquid medium and rendered lower dry weight values than the isogenic strain, *S*. *lividans* TK21 (pIJ486) (Fig 1a), while the alpha-amylase overproducer strain grew in a more dispersed manner rendering higher dry weight values than the isogenic strain *S*. *lividans* TK21 (pIJ486).

*S*. *lividans* does not sporulate when grown in liquid medium, but differences in growth of the overproducer strains seemed to be reflected in sporulation (S1 Fig). The alpha-amylase overproducer strain showed a delayed sporulation phenotype, a characteristic previously described in *B*. *subtilis* overproducing alpha-amylase [11].

The effect of the overproduced model enzymes on the overall gene expression of the respective bacterial cells was assessed using hybridisation to genome-wide microarrays. Total RNA was extracted from the cell cultures in liquid minimal medium at the late exponential phase of growth. All microarray analyses were performed on RNA samples obtained from three independent cultures grown under identical conditions.

The cDNA obtained from each RNA preparation of the overproducer strains was hybridised to the cDNA obtained from the equivalent RNA preparations of its isogenic strain, *S*. *lividans* (pIJ486). Thresholds of probability values (*p* values) below 0.05 and fold change above 2 or below -2 were used to select differential hybridisation spot results. The results obtained for the alpha-amylase and agarase overproducer strains at the late exponential phase of growth are summarised in Tables 1 and 2, respectively.

**Table 1:** Genes modulated by alpha-amylase propagation in high copy number.

Gene	Transcriptional unit	Annotated function	Ratio pAMI11/pIJ486
***UPREGULATED***			
*Central carbon metabolism*			
SLI2180	SLI2180-2181	*pdhL* putative dihydrolipoamide dehydrogenase	2.29
SLI4808	SLI4808-4809	*sucC*, succinyl-CoA synthetase beta chain	3.19
*ABC transporters*			
SLI2231		*malE*, putative maltose-binding protein	2.095
SLI5774	SLI5777-5774	*gluD* glutamate permease	3.12
SLI5775	SLI5777-5774	*gluC* glutamate permease	2.97
SLI5776	SLI5777-5774	*gluB*, glutamate binding protein	2.29
SLI5777	SLI5777-5774	*gluA*, glutamate uptake system ATP-binding	2.18
*Oxidative phosphorylation*			
SLI2151	SLI2151-2148	*cox* cytochrome c oxidase subunit III	2.26
SLI2155	SLI2156-2153	*cox1* possible cytochrome c oxidase subunit I	2.62
SLI5369	SLI5367-5374	*atpf*, ATP synthase B chain	2.08
*Aminoacids metabolism*			
SLI4645	SLI4645	*aspC*, aspartate aminotransferase	2.04
*Purine/ Pyrimidine metabolism*			
SLI4654	SLI4654-4655	*rpoB*, DNA-directed RNA polymerase beta chain	2.18
SLI5805	SLI5805	*nrdJ*, ribonucleotide reductase	3.14
*Oxidative stress*			
SLI0999	SLI0999	*sodF*2, superoxide dismutase	2.71
SLI2633	SLI2633	*sodF*, superoxide dismutase [Fe-Zn]	2.55
SLI5254	SLI5254	*sodN*, superoxide dismutase	2.15
*Secreted proteins*			
SLI5029	SLI5029-5030	Putative secreted protein	2.27
SLI6108	SLI6108-6109	*fusH*, esterase	2.28
SLI7019	SLI7019	*aml*, secreted alpha-amylase	2.31
SLI7020	SLI7020	*amlB*, secreted alpha-amylase	2.73
*Ribosomal function & biogenesis*			
SLI1321	SLI1321	*tuf3*, elongation factor TU-3	2.70
SLI1598	SLI1600-1596	*rplT* 50 S ribosomal protein L20	2.55
SLI1599	SLI1600-1596	*rpmI*, 50 S ribosomal protein L35	3.15
SLI2597	SLI2597-2595	*rplU*, ribosomal protein L21	2.35
SLI3425	SLI3425	*rpsR2* possible 30S ribosomal protein S18	3.36
SLI3906	SLI3906	*rpsF*, putative 30 S ribosomal protein S6	2.65
SLI3908	SLI3908-3909	*rpsR* possible ribosomal protein S18	2.62
SLI4648	SLI4646-4649	*rplK*, ribosomal protein L11	3.03
SLI4652	SLI4652-4653	*rpIJ* 50S ribosomal protein L10	2.01
SLI4653	SLI4652-4653	*rpIL*, 50S ribosomal protein L7/L12	2.63
SLI4659	SLI4659-4662	*rpsL* 30S ribosomal protein S12	2.02
SLI4660	SLI4659-4662	*rpsG* 30S ribosomal protein S7	3.82
SLI4661	SLI4659-4662	*fusA*, elongation factor G	2.13
SLI4662	SLI4659-4662	*tuf1*, elongation factor TU-1	2.45
SLI4703	SLI4701-4721	*rplD* 50S ribosomal protein L4	2.68
SLI4706	SLI4701-4721	*rpsS* 30S ribosomal protein S19	2.57
SLI4708	SLI4701-4721	*rpsC* 30S ribosomal protein S3	2.75
SLI4709	SLI4701-4721	*rplP* 50S ribosomal protein L16	2.78
SLI4710	SLI4701-4721	*rpmC* 50S ribosomal protein L29	3.76
SLI4712	SLI4701-4721	*rplN* 50S ribosomal protein L14	2.97
SLI4714	SLI4701-4721	*rplE* 50S ribosomal protein L5	3.91
SLI4715	SLI4701-4721	*rpsN* 30S ribosomal protein S14	3.26
SLI4717	SLI4701-4721	*rplF* 50S ribosomal protein L6	3.98
SLI4719	SLI4701-4721	*rpsE* 30S ribosomal protein S5	2.12
SLI4720	SLI4701-4721	*rpmD* 50S ribosomal protein L30	3.10
SLI4721	SLI4701-4721	*rplO* 50S ribosomal protein L15	2.39
SLI4725	SLI4724-4731	*infA* translational initiation factor IF1	3.97
SLI4726	SLI4724-4731	*rpmJ* 50S ribosomal protein L36	2.74
SLI4727	SLI4724-4731	*rpsM* 30S ribosomal protein S13	2.35
SLI4728	SLI4724-4731	*rpsK* 30S ribosomal protein S11	2.09
SLI4730	SLI4724-4731	*rplQ*, 50S ribosomal protein L17	2.52
SLI4734	SLI4734-4735	*rplM* 50S ribosomal protein L13	2.82
SLI4735	SLI4734-4735	*rpsI*, 30S ribosomal protein S9	2.3
SLI5359	SLI5359-5362	*rpmE3* 50S ribosomal protein	2.13
*Others*			
SLI1515	SLI1515-1516	*secF*, protein-export membrane protein	2.03
SLI1547	SLI1545-1547	Possible anthranilate synthase	2.03
SLI1637	SLI1634-1637	Conserved hypothetical protein	2.28
SLI3793	SLI3793	Conserved hypothetical protein	2.14
SLI4252	SLI4251-4253	Hypothetical protein	3.08
SLI4253	SLI4251-4253	Conserved hypothetical protein	3.22
SLI4822	SLI4822	Possible integral membrane protein	2.19
SLI5271	SLI5271	Conserved hypothetical protein	2.24
SLI6148	SLI6148	Hypothetical protein	2
SLI6986	SLI6986	Possible DNA-binding protein	2.03
SLI7713	SLI7713	Hypothetical protein	2.39
***DOWNREGULATED***			
*Morphological differentiation*			
SLI4002	SLI4002	*nepA*, possible secreted protein	-2.135
*Others*			
SLI1604	SLI1604	Conserved hypothetical protein	-2.05
SLI5028	SLI5028	Possible ATP-binding protein	-2.6

**Table 2:** Genes modulated by agarase propagation in high copy number.

Gene	Transcriptional unit	Annotated function	Ratio pAGAs5/pIJ486
***UPREGULATED***			
*Aminoacids catabolism*			
SLI2779	SLI2776-2779	*acdH*, acyl-CoA dehydrogenase	2.15
SLI5676	SLI5676	*gabT* possible 4-aminobutyrate aminotransferase	2.39
*Morphological differentiation*			
SLI4002	SLI4002	*nepA*, putative secreted protein	2.76
SLI4184	SLI4184	*amfC*, aerial mycelium formation	2.18
SLI6682	SLI6681-6684	*ramS*, hypothetical protein	3.34
*Secreted proteins*			
SLI3471	SLI3471	*dagA*, extracellular agarase precursor	3.07
*Transcriptional regulators*			
SLI3328	SLI3328-3327	*bdtA*, hypothetical protein	2.1
*Others*			
SLI0057	SLI0057	Hypothetical protein	2.17
SLI0141	SLI0141	Possible calcium-binding protein	2.39
SLI0210	SLI0210	Hypothetical protein	2.26
SLI0315	SLI0315	Possible decarboxylase	2.45
SLI0316	SLI0316	Hypothetical protein	2.04
SLI0679	SLI0679	Hypothetical protein	2.09
SLI1640	SLI1641-1640	Conserved hypothetical protein	2.94
SLI3009	SLI3009	Hypothetical protein	2.47
SLI3472	SLI3472	Possible transposase remnant	2.62
SLI4440	SLI4440	Hypothetical protein	3.31
SLI5028	SLI5028	Possible ATP-binding protein	3.19
SLI6030	SLI6030	Conserved hypothetical protein	2.18
SLI6869	SLI6869	Hypothetical protein	2.37
SLI7647	SLI7647	Putative calcium binding protein	2.10
***DOWNREGULATED***			
*Nitrogen metabolism*			
SLI0218	SLI0216-0219	*narJ2*, nitrate reductase delta chain NarJ2	-2.19
SLI0219	SLI0216-0219	*narJ2*, nitrate reductase delta chain NarI2	-2.36
*Central carbon metabolism*			
SLI2180	SLI2180-2181	*pdhL* putative dihydrolipoamide dehydrogenase	-2.11
SLI4808	SLI4808-4809	*sucC*, succinyl-CoA synthetase beta chain	-3.04
SLI4858	SLI4858-4855	*dhsC*, possible succinate dehydrogenase membrane subunit	-2.13
*ABC transporters*			
SLI5774	SLI5777-5774	*gluD* glutamate permease	-2.83
SLI5775	SLI5777-5774	*gluC* glutamate permease	-2.93
SLI5776	SLI5777-5774	*gluB*, glutamate binding protein	-2.40
SLI5777	SLI5777-5774	*gluA*, glutamate uptake system ATP-binding	-2.44
*Oxidative phosphorylation*			
SLI2150	SLI2151-2148	*qcrC* cytochrome C heme-binding subunit	-2.77
SLI2151	SLI2151-2148	*cox3* cytochrome c oxidase subunit III	-3.46
SLI2155	SLI2156-2153	*cox1* possible cytochrome c oxidase subunit I	-2.47
SLI2156	SLI2156-2153	*cox*, possible cytochrome c oxidase subunit II	-2.7
SLI3945	SLI3945-3947	*cydA*, cytochrome oxidase subunit I	-2.03
SLI5366	SLI5366	*atpI*, ATP synthase protein I	-2.14
SLI5367	SLI5367-5374	*atpB*, ATP synthase A chain	-2.1
SLI5368	SLI5367-5374	*atpE*, ATP synthase C chain	-2.61
SLI5369	SLI5367-5374	*atpf*, ATP synthase B chain	-2.92
SLI5370	SLI5367-5374	*atpH*, ATP synthase delta chain	-2.18
SLI5371	SLI5367-5374	*atpA*, ATP synthase alpha chain	-2.46
SLI5372	SLI5367-5374	*atpG*, ATP synthase gamma chain	-2.79
*Aminoacids metabolism*			
SLI1773	SLI1773	Putative L-alanine dehydrogenase	-2.21
*Fatty acids biosynthesis*			
SLI2389	SLI2387-2390	*acpP*, acyl carrier protein	-2.44
SLI2390	SLI2387-2390	*fabF*, 3-oxoacyl carrier [acyl-carrier-protein synthase II]	-2.31
*Purine/ Pyrimidine metabolism*			
SLI4654	SLI4654-4655	*rpoB*, DNA-directed RNA polymerase beta chain	-2.16
*Ribosomal function & biogenesis*			
SLI1491	SLI1491-1490	elongation factor P	-2.08
SLI1598	SLI1600-1596	*rplT* 50 S ribosomal protein L20	-2.44
SLI1599	SLI1600-1596	*rpmI*, 50 S ribosomal protein L35	-3.7
SLI1600	SLI1600-1596	*infC*, putative translation initiation factor IF-3	-2.42
SLI1998	SLI1998	*rpsA*, 30S ribosomal protein S1	-2.56
SLI2597	SLI2597-2595	*rplU*, ribosomal protein L21	-3.21
SLI3425	SLI3425	*rpsR2* possible 30S ribosomal protein S18	-2.29
SLI3906	SLI3906	rpsF, putative 30S ribosomal protein S6	-3.76
SLI3908	SLI3908-3909	*rpsR* possible ribosomal protein S18	-2.6
SLI4648	SLI4646-4649	*rplK*, ribosomal protein L11	-4.98
SLI4649	SLI4646-4649	*rlpA*, 50S ribosomal protein L1	-2.44
SLI4652	SLI4652-4653	*rpIJ* 50S ribosomal protein L10	-2.58
SLI4659	SLI4659-4662	*rpsL* 30S ribosomal protein S12	-3.52
SLI4660	SLI4659-4662	*rpsG* 30S ribosomal protein S7	-4.66
SLI4661	SLI4659-4662	*fusA*, elongation factor G	-2.31
SLI4701	SLI4701-4721	*rpsJ*, 30 S ribosomal protein S10	-3.72
SLI4702	SLI4701-4721	*rplC*, 50 S ribosomal protein L3	-2.58
SLI4703	SLI4701-4721	*rplD* 50S ribosomal protein L4	-2.90
SLI4704	SLI4701-4721	*rplW*, 50S ribosomal protein L23	-3.16
SLI4705	SLI4701-4721	*rplB*, 50S ribosomal protein L2	-3.39
SLI4706	SLI4701-4721	*rpsS*, 30S ribosomal protein S19	-6.11
SLI4707	SLI4701-4721	*rplV*, 50S ribosomal protein L22	-4.87
SLI4708	SLI4701-4721	*rpsC*, 30S ribosomal protein S3	-4.28
SLI4709	SLI4701-4721	*rplP*, 50S ribosomal protein L16	-4.43
SLI4710	SLI4701-4721	*rpmC*, 50S ribosomal protein L29	-3.19
SLI4712	SLI4701-4721	*rplN*, 50S ribosomal protein L14	-4.06
SLI4713	SLI4701-4721	*rplX*, 50S ribosomal protein L24	-3.68
SLI4714	SLI4701-4721	*rplE*, 50S ribosomal protein L5	-5.54
SLI4715	SLI4701-4721	*rpsN*, 30S ribosomal protein S14	-4.46
SLI4716	SLI4701-4721	*rpsH*, 30S ribosomal protein S8	-3.96
SLI4717	SLI4701-4721	*rplF*, 50S ribosomal protein L6	-4.2
SLI4718	SLI4701-4721	*rplR*, 50S ribosomal protein L18	-2.52
SLI4719	SLI4701-4721	*rpsE* 30S ribosomal protein S5	-2.8
SLI4720	SLI4701-4721	*rpmD*, 50S ribosomal protein L30	-2.94
SLI4721	SLI4701-4721	*rplO*, 50S ribosomal protein L15	-2.14
SLI4725	SLI4724-4731	*infA*, translational initiation factor IF1	-4.59
SLI4726	SLI4724-4731	*rpmJ*, 50S ribosomal protein L36	-3.49
SLI4727	SLI4724-4731	*rpsM*, 30S ribosomal protein S13	-3.34
SLI4728	SLI4724-4731	*rpsK*, 30S ribosomal protein S11	-2.66
SLI4729	SLI4724-4731	*rpoA*, DNA-directed RNA polymerase alpha chain	-2.34
SLI4730	SLI4724-4731	*rplQ*, 50S ribosomal protein L17	-2.51
SLI4734	SLI4734-4735	*rplM*, 50S ribosomal protein L13	-4.22
SLI4735	SLI4734-4735	*rpsI*, 30S ribosomal protein S9	-3.76
SLI5571	SLI5570-5572	50S ribosomal protein L32	-2.21
SLI5595	SLI5593-5595	*rplS*, 50S ribosomal protein L19	-2.13
SLI5624	SLI5624-5625	*rpsB*, 30S ribosomal protein S2	-2.73
*Secreted proteins*			
SLI5029	SLI5029-5030	Possible secreted protein	-3.41
*Others*			
SLI0207	SLI0206-0207	Conserved hypothetical protein	-2.26
SLI4657	SLI4657	Possible integral membrane protein	-2.1
SLI5650	SLI5650	Possible membrane protein	-2.53
SLI7428	SLI7427-7428	*hmpA1*, possible flavohemoprotein	-2.77

Hybridisation data from RNA extracted at the early stationary phase of growth were very dispersed (not shown), probably due to the bacterial heterogeneity, as observed previously [12, 13].

Sixty-five genes including the alpha-amylase gene (*amlB*) encoding AmlB, were upregulated in the alpha-amylase overproducer strain and only three genes were downregulated, while in the case of the agarase overproducer strain twenty-one genes, including the agarase gene (*dagA*) encoding DagA, were upregulated and seventy-six genes were downregulated.

When the transcriptional profiles of the overproducer strains were compared, forty-one upregulated genes (of the 65 upregulated ones) in the alpha-amylase overproducer strain were downregulated (of the 76 downregulated ones) in the agarase overproducer strain. The validity of the results was analysed by quantitative RT-PCR of some of the opposite coinciding regulated genes (Table 3). The most abundant functional group of these 41 genes consisted of the ribosomal genes. Apart from the ribosomal genes, other genes seem to be associated with active cell growth, that is, carbon metabolism, oxidative phosphorylation, purine / pyrimidine biosynthesis and the glutamate ABC transporter (Table 3).

**Table 3:** Opposite regulated genes upregulated in alpha-amylase overproduction and downregulated in agarase overproduction.

Gene	Ratio pAMI11/pIJ486		Ratio pAGAs5/pIJ486		Transcriptional unit	Annotated function
	Ratio arrays	Ratio qRTPCR	Ratio arrays	Ratio qRTPCR		
*Central carbon metabolism*						
SLI2180	2.29		-2.11		SLI2180-2181	*pdhl*, putative dihydrolipoamide dehygrogenase
SLI4808	3.19	8.74 ± 0.12	-3.04	-3.85± 0.12	SLI4808-4809*	*sucC*, succinyl-CoA synthetase beta chain
*ABC transporters*						
SLI5774	3.12		-2.83		SLI5777-5774*	*gluB*, glutamate permease
SLI5775	2.97		-2.93		SLI5777-5774*	*gluC*, glutamate permease
SLI5776	2.29	5.78 ± 2.65	-2.40	-8.92 ± 0.87	SLI5777-5774*	*gluB*, glutamate binding protein
SLI5777	2.18		-2.44		SLI5777-5774*	*gluA*, glutamate uptake system ATP- binding
*Oxidative phosphorylation*						
SLI2151	2.26	3.32 ± 1.5	-3.46	-3.68 ± 0.55	SLI2151-2148*	*cox* cytochrome *c* oxidase subunit III
SLI2155	2.62		-2.47		SLI2156-2153	*cox*1 possible cytochrome *c* oxidase subunit I
SLI5369	2.08	3.45 ± 1.37	-2.92	-7.66 ± 4,59	SLI5367-5374*	*atpf*, ATP synthase B chain
*Purine and pyrimidine metabolism*						
SLI4654	2.18		-2.16		SLI4654-4655	*rpoB*, DNA-directed RNA polymerase beta chain
*Ribosomal function & biogenesis*						
SLI1598	2.55		-2.44		SLI1600-1596*	*rplT* 50S ribosomal protein L20
SLI1599	3.15		-3.7		SLI1600-1596*	*rpmI*, 50 S ribosomal protein L35
SLI2597	2.35		-3.21		SLI2597-2595	*rplU*, ribosomal protein L21
SLI3425	3.36		-2.29		SLI3425	*rpsR2* possible 30S ribosomal protein S18
SLI3906	2.65		-3.76		SLI3906	*rpsF*, putative 30S ribosomal protein S6
SLI390	2.62		-2.6		SLI3908-3909*	*rpsR* possible ribosomal protein S18
SLI4648	3.03		-4.98		SLI4646-4649*	*rplK*, ribosomal protein L11
SLI4652	2.01		-2.58		SLI4652-4653	*rpIJ* 50S ribosomal protein L10
SLI4659	2.02		-3.52		SLI4659-4662*	*rpsL* 30S ribosomal protein S12
SLI4660	3.82		-4.66		SLI4659-4662*	*rpsG* 30S ribosomal protein S7
SLI4661	2.13		-2.31		SLI4659-4662*	*fusA*, elongation factor G
SLI4703	2.68		-2.90		SLI4701-4721*	*rplD* 50S ribosomal protein L4
SLI4706	2.57		-6.11		SLI4701-4721*	*rpsS*, 30S ribosomal protein S19
SLI4708	2.75		-4.28		SLI4701-4721*	*rpsC*, 30S ribosomal protein S3
SLI470	2.78		-4.43		SLI4701-4721*	*rplP*, 50S ribosomal protein L16
SLI4710	3.76		-3.19		SLI4701-4721*	*rpmC*, 50S ribosomal protein L29
SLI4712	2.97		-4.06		SLI4701-4721*	*rplN*, 50S ribosomal protein L14
SLI4714	3.91		-5.54		SLI4701-4721*	*rplE*, 50S ribosomal protein L5
SLI4715	3.26		-4.46		SLI4701-4721*	*rpsN*, 30S ribosomal protein S14
SLI4717	3.98		-4.2		SLI4701-4721*	*rplF*, 50S ribosomal protein L6
SLI4719	2.12		-2.8		SLI4701-4721*	*rpsE* 30S ribosomal protein S5
SLI4720	3.10		-2.94		SLI4701-4721*	*rpmD*, 50S ribosomal protein L30
SLI4721	2.39	13.72 ± 4.56	-2.14	-7.31 ± 4.56	SLI4701-4721*	*rplO*, 50S ribosomal protein L15
SLI4725	3.97		-4.59		SLI4724-4731*	*infA*, translational initiation factor IF1
SLI4726	2.74		-3.49		SLI4724-4731*	*rpmJ*, 50S ribosomal protein L36
SLI4727	2.35	6.64 ± 2.75	-3.34	-4.3 ± 0.72	SLI4724-4731*	*rpsM*, 30S ribosomal protein S13
SLI4728	2.09		-2.66		SLI4724-4731*	*rpsK*, 30S ribosomal protein S11
SLI4730	2.52		-2.51		SLI4724-4731*	*rplQ*, 50S ribosomal protein L17
SLI4734	2.82		-4.22		SLI4734-4735*	*rplM*, 50S ribosomal protein L13
SLI4735	2.3		-3.76		SLI4734-4735*	*rpsI*, 30S ribosomal protein S9
*Secreted proteins*						
SLI5029	2.27		-3.41		SLI5029-5030	Possible secreted protein

*Transcriptional units potentially ppGpp regulated in *S*. *coelicolor* [11]

In *Streptomyces* the downregulation of ribosomal genes has been reported to form part of the so-called “stringent response” [14] where RelA appears to be the only regulator for the ppGpp synthesis, the stringent response alarmone [15]. The expression of *relA* was measured by qRT-PCR analysis in the overproducer strains with respect to the isogenic strain at the late exponential phase of growth. The gene *relA* was upregulated in the agarase overproducer strain (2.69 ± 0.19), while in the alpha-amylase overproducer strain the relative expression level of *relA* appeared to be on the same level as that of the isogenic strain (1.33 ± 0.24).

### The Tat route is mainly expressed at the late phase of growth

The synthesis and secretion of the AmlB and DagA in the corresponding overproducer strain were monitored by Western blot analysis. To correct the biomass differences between the overproducer strains, samples have been loaded normalised by the same amount of bacterial culture dry weight. No precursor or mature forms of the two model proteins were revealed by the anti-AmlB or anti-DagA serum to be associated to the cellular fraction of the overproducer strains, strongly suggesting that synthesis, transport and secretion of alpha-amylase and agarase took place very efficiently in the overproducer strains. No alpha-amylase or agarase were detected in the *S*. *lividans* TK21 (pIJ486) strain harbouring the vector with no cloned genes and carried out as a negative control (not shown).

The maximum level of active mature AmlB appeared at the late exponential phase of growth and decreased when the culture reached the stationary phase (Fig 1b and 1d, [16]), while in the case of DagA the mature protein started to accumulate extracellularly mainly at the early stationary phase, reaching its maximum at the late phase of growth (Fig 1c and 1e, [10]). In good agreement with this, the expression of *tatC*, the gene encoding TatC, one of the Tat complex components, attained its maximum expression level at the stationary phase, as determined by qRT-PCR analysis (relative *tatC* expression levels at the late exponential phase, early stationary and late stationary phase of growth were 0.41± 0.16, 2.91± 0.63 and 3.38 ± 0.35, respectively). Relative differences in the yield of each enzyme are dependent on the specific reaction with their respective antibodies, which would probably bias a potential yield comparative analysis.

## Discussion

*S*. *lividans* has been tested as a host for a wide variety of proteins encoded by genes from different origins, and the varying yields obtained depended on the protein overproduced. In some cases a high yield of a particular protein in its native conformation was obtained [3], while in others there was a much lower amount of correctly folded protein. Therefore, the improvement in secreted protein yield is something that deserves to be investigated. Classic attempted approaches consisted of codon usage optimization, the use of strong and controllable promoters, use of high copy plasmids to propagate the genes, use of efficient signal peptides and the overexpression of some components of the protein secretion pathway [17]. Transcriptomic studies at the cellular level, comparing the wild type and the overproducer strain, could shed some light on the overall bacterial gene expression pattern fluctuations, which eventually may lead to a notable improvement in the desired protein produced by the cell. Other authors have engineered the overproduction of secretory proteins in *S*. *lividans* in a different way to ours; however, transcriptomic analyses were not available in these studies [18,19]. This is an area where sparse knowledge has been generated to date and deserves further attention [17].

The results obtained encouraged us to continue exploring so as to potentially improve extracellular protein overproduction in streptomycetes.

*Streptomyces* uses two routes to secrete proteins: the major secretory route (Sec pathway), which secretes proteins in an unfolded manner and the minor secretory route (Tat pathway) that enables the secretion of the protein in a folded conformation. Alpha-amylase (a Sec-dependent protein, [8]) and agarase (a Tat-dependent protein, [9]) were used as model proteins in our experiments to assess the influence of their respective overproduction on the overall bacterial gene expression pattern when compared to the isogenic strain.

A comparison of the transcriptomic results obtained in each case showed a number of opposite regulated genes pertaining mainly to the stringent response, which were upregulated in the alpha-amylase overproducer strain and downregulated in the agarase overproducer strain, the ribosomal genes being the main functional group within these genes (Table 3). Accordingly, the *relA* expression was unaffected in the alpha-amylase overproducer strain and upregulated in the agarase overproducer strain when compared with the isogenic strain. The opposite regulated genes are also related to active cell growth (central carbon metabolism, oxidative phosphorylation, purine and pyrimidine metabolism, and ribosomal genes) (Table 3).

Additionally, glutamate and aspartate are taken up rapidly by the *S*. *lividans* strains and are known to be preferential carbon and energy sources when bacteria are cultivated in minimal liquid medium supplemented with casamino acids [20]. Interestingly enough, in the agarase overproducer strain, genes related to glutamate uptake were downregulated according to a stringent response situation [14] while in the case of the alpha-amylase overproducer strain these were upregulated (Table 3), thus showing the effect of protein overproduction on the overall regulation of the bacterial carbon metabolism.

Hence, the growth in liquid medium of the overproducer strains is different. The alpha-amylase overproducer strain grows better than the isogenic strain (Fig 1a) while the agarase overproducer strain shows a relatively reduced growth and formation of denser clumps with respect to the isogenic strain (Fig 1a). The alpha-amylase overproducer strain showed much vigorous cell growth and may need a higher level of expression of genes encoding ribosomal rRNA and ribosomal components as well as additional carbon source transporters.

The stringent response is induced in *Streptomyces* via the depletion of amino acids [14]. This response occurs naturally in the isogenic *S*. *lividans* strain at the early stationary phase of growth (qRT-PCR relA 5.69 ± 2.83) yet not at earlier phases of growth where nutrients are still in excess.

Stringent response is also induced when the solute binding proteins necessary for nutrients’ uptake are absent [21]. It has been observed that major secretory protein overproduction, constituted mainly of Sec proteins, was triggered by the presence in high copy number of a two-component system regulator in *S*. *coelicolor* [13], as well as the induction of the stringent response, strongly suggesting that when the cell detects nutrient depletion (amino acids by protein overproduction), the stringent response is triggered.

The overproducer strains contain *amlB* and *dagA* coding sequences under the control of their regulatory region and were cloned into the multicopy plasmid pIJ486 to maximise their relative expression level. The size of the overproduced model proteins does not appear to be related to possible amino acids depletion due to the overproduction, since AmlB has a larger size (59kDa) than DagA (32kDa), and its overproduction does not cause the stringent response. The overall amino acids composition is similar in both proteins, and it seems unlikely that this could be responsible for any particular amino acid depletion.

However, the fact that the Tat route is functional at a late phase of growth suggests that the overproduction of the Tat-dependent model protein (agarase), which reached its maximum presence at the supernatant during that phase may further aggravate the cellular stress, causing a greater amino acids depletion, thus eliciting the stringent response, something that does not occur when alpha-amylase is overproduced and secreted via the Sec route, reaching its maximum extracellular presence at the exponential phase of growth.

Alpha-amylase overproduction triggers the *S*.*lividans* CssRS two-component system, which regulates the synthesis of three proteases that specifically degrade misfolded proteins [16]. This system is not active when agarase is overproduced in *S*. *lividans* [16]. The degradation of misfolded proteins could provide supplementary amino acids to the medium, which in turn favours the absence of a stringent response and probably contributes to the upregulation of genes related to the active cell growth.

This potential supplementary source of nutrients could contribute to the downregulation of genes involved in the morphological differentiation in *Streptomyces*, whose expression is thought to be triggered by nutritional limitations [22], which eventually may explain the deficiency in sporulation observed in the alpha-amylase overproducer when grown in solid medium.

The *Streptomyces* Tat system deserves to be studied further as regards its possible exploitation for secretory protein production, since the production of extracellular proteins apparently appears in the supernatants in a folded active conformation. Nevertheless, this system leads to a potential depletion of precursors, while engineering the secretion of extracellular proteins via the Sec route ensures an efficient secretion of proteins, apparently causing no metabolic damage to the cell.

The obtained results revealed contrary *S*. *lividans* responses to the stress induced by the overproduction of a Sec or a Tat model protein. These responses signalled possible drawbacks in the protein production process that would have to be taken into account to improve secretory protein yields. The induction of the stringent response when the Tat model protein is overproduced could reduce the expected yield, as the stringent response may provoke cellular death, which is a potential drawback that has to be considered when designing secretory protein production processes. Thus, the Sec pathway could be the route of choice when engineering extracellular secretory protein production in *Streptomyces*.

To our knowledge, this work is the first transcriptomic approach to studying the different bacterial responses to the overproduction of a Sec and a Tat model secretory protein. The overproduction of other known secretory proteins in *Streptomyces* would be needed in order to more precisely draw reproducible bacterial response patterns to secretory protein overproduction.

Moreover, our transcriptional studies have revealed a set of genes, differentially regulated for either the Sec or the Tat route that could be useful markers to monitor scale-up secretory production processes in *S*. *lividans*.

## Methods

### Bacterial strains, plasmids and media

*S*. *lividans* TK21, a non-plasmid derivative from *Streptomyces lividans 66* (John Innes Center Collection, Norwich UK) was a generous gift from the D. A. Hopwood and was used as the wild type strain.

Plasmid pAMI11 [8] is a pIJ486 [23] derivative and plasmid pAGAs5 [10] is a pAGAs1 [24] derivative, carrying the *S*. *lividans amlB* and the *S*. *coelicolor* agarase (*dagA*) coding sequences and regulatory regions respectively, as well as a frame-shift-mutated thiostrepton resistance gene.

Plasmids pIJ486 were used to transform *S*. *lividans* TK21protoplasts to generate the corresponding strains. *S*. *lividans* TK21 (pIJ486) was used as the isogenic strain.

The strains were cultured in minimal liquid medium (NMMP): 1% mannitol, 2 g/L (NH_4_)_2_ SO_4_, 5 g/L Bacto casamino acids, 0.6 g/L MgSO_4_.7 H_2_O, 6.8 g/L Na H_2_PO_4_, 11.4 g/L K_2_HPO_4_ and 1 ml/L minor elements solution (containing 1 g/L ZnSO_4_. 7 H_2_O, 1 g/L FeSO_4_.7H_2_O, 1g/L MnCl_2_. 4 H_2_O and 1 g/L anhydrous CaCl_2_) and solid MS and R5 medium [25]. Thiostrepton (50 μg ml^-1^) and kanamycin (50 μg ml^-1^) were added to the solid media when required. *E*. *coli* DH5α was cultured in Luria broth (LB) [26].

### RNA extraction, labelling and hybridisation

Total RNA was isolated from 50 ml aliquots of bacteria-growing NMMP cultures in Erlenmeyer flasks at 30°C with continuous shaking at 250 rpm at the late exponential phase of growth (approx. 24 h of growth) using the RNeasy midi Kit (Qiagen). Cell lysates were extracted twice with phenol-chloroform before being loaded onto RNeasy midi columns for RNA purification.

Fluorescently labelled cDNA for microarray hybridisation was obtained using the SuperScript Indirect cDNA Labelling System (Invitrogen), following the supplier’s instructions. Twenty micrograms of RNA were transformed to cDNA with Superscript III reverse transcriptase using random hexamers as primers, adding aminoallyl-modified nucleotides to the reaction mixture. After cDNA purification, the Cy3 or Cy5 fluorescent dyes (Invitrogen) were coupled to the amino-modified first-strand cDNA. Labelling efficiency was assessed using a NanoDrop ND1000 spectrophotometer (NanoDrop Technologies). Prior to the hybridisation process, *Streptomyces coelicolor* genome-wide DNA microarrays (Eurogentec, Belgium) were blocked by immersion into a 50 ml Falcon tube containing 5xSSC, 0.1% (w/v) SDS and 1% (w/v) bovine serum albumin, and preheated to 42°C. After 45 min at 42°C, the microarrays were washed by being briefly immersed in a Falcon tube containing sterile water at room temperature, followed, when necessary, by another immersion in isopropanol, before being allowed to dry.

Equal amounts of Cy3- or Cy5-labelled cDNAs (about 50 pmoles each), one sample corresponding to the control and the other to the problem under analysis, were mixed and dried in a Speed-Vac. Each sample was dissolved in 45 ml of a solution containing 50% (v/v) deionised formamide, 5 x Denhardt’s solution, 6 x SSC, 0.5% (w/v) SDS, 5% (w/v) dextran sulphate, pre-filtered and pre-heated at 42°C. After 2 min at 90°C to denature the DNA, the solution was applied to the microarray slide and covered with a 24 x 60 mm cover glass. The slide was introduced into a hybridisation chamber and incubated for 18 h away from the light, following the microarray supplier’s instructions. The microarray was then transferred to a Falcon tube containing 0.5 x SSPE (1 x SSPE contains 150 mM NaCl, 1 mM EDTA, 11.5 mM NaH_2_PO_4_, PH 7.4), 0.5% (w/v) SDA and pre-heated to 37°C. After removing the cover glass, the microarray was washed by gentle shaking for 5 min. The slide was subsequently transferred to a new tube containing 0.5 x SSPE and 0.5% (w/v) SDS, and washed again by gentle shaking for 5 min at room temperature. Similar washes with 0.5 x SSPE were conducted three more times, followed by a final wash with 0.1 x SSPE at room temperature. The microarray was allowed to dry and scanned in a microarray scanner with green and red lasers operating at 532 and 635 nm, respectively, to excite the Cy3 and Cy5. Images were taken at 10 mm resolution and spot intensity was determined using the Genepix Pro 5.0 (Axon) software package.

Hybridisation data were statistically analysed using LIMMA [27] software. Three independent RNA extractions were conducted out for each experiment, the corresponding microarray analyses were performed and the information was provided by three biological replicas combined in each case. The results for each replica (median intensity for each channel) were normalised and statistically analysed using the LIMMA software package [27]. Background subtraction was performed using a method implemented in LIMA designed to yield positive corrected intensities (i.e. to avoid negative intensity values). A convolution of normal and exponential distributions was fitted to the foreground intensities using the background intensities as covariate. This resulted in a smooth monotonic transformation of the background-subtracted intensities in such a way that all the corrected ones were positive. Differential hybridisation was calculated using linear models and empirical Bayes moderated *t*-statistics [27, 28]. The resulting log-ratios were normalised for each array through print-tip loess [28] and differential hybridisation values were scaled to achieve consistency among arrays. Each probe was tested for changes in differential hybridisation over replicates by using moderated *t*-statistics [27]. The p-values were adjusted for multiple testing, as described [29], to control the false discovery rate. The output file provided the fold-change and p-values for each spot, among other data. Comparisons were performed using the Venn algorithm (http://bioinfogp.cnb.csic.es/tools/venny/index.html.) (http://www.pangloss.com/seidel/Protocols/venn.cgi). Operon prediction was carried out using the Microbesonline website (http://microbesonline.org).

### Microarray data accession number

The microarray data presented in this paper have been registered in the NCBIGEO data bank (http://www.ncbi.nlm.nih.gov/geo: accession number GSE61447- GSE61448).

### Quantitative real time PCR (qRT-PCR)

Total RNA was isolated from bacteria-growing cultures using the RNeasy midi Kit (Qiagen). Cell lysates were extracted twice with phenol-chloroform before being loaded onto RNeasy midi columns for RNA purification. DNA potentially contaminating the RNA preparations was removed by incubation with RNase-free DNAse (Ambion) and its absence was tested by quantitative real time PCR amplification in the absence of reverse transcriptase. Complementary DNA was synthesised using the High Capacity Archive kit (Applied Biosystems). Quantitative real time PCR (qRT-PCR) was performed using SYBR Green technology in an ABI Prism 7300 Sequence Detection System (Applied Biosystems). Samples were initially denatured by heating at 95°C for 10 min. A 40-cycle amplification and quantification program was then followed (95°C for 15 sec and 60°C for 1 min) by a single fluorescence measurement per cycle, in accordance with the manufacturer’s recommendations. Three biological samples from the different bacterial cultures were amplified in triplicate in separate PCR reactions. All PCR products were between 50 and 150 bp in length.

A melting curve analysis was conducted after amplification to distinguish the targeted PCR products from the non-targeted ones. The melting curves were obtained by heating at temperatures ranging from 60°C to 95°C at a rate of 0.2°C per sec, with continuous fluorescence scanning. The raw threshold cycle (C_T_) values were converted to relative expression levels by the 2 ^-ΔΔ CT^ method [30] to quantify the relative gene expression.

Oligonucleotides HRDBD (5’-GGACAAGCTGGCGAACTC -3’) and HRDBR (5’-CCTCCAGCAGGTGGTTCT -3’) were used to amplify the *hrdB* transcript carried out as an internal control to quantify the relative expression of the target genes. The oligonucleotides used as primers to amplify other transcripts are indicated in S1 Table.

### Protein analysis and Western blot experiments

Standard extracellular protein analyses were essentially carried out as described [31]. Supernatants from cells grown in NMMP medium were collected by centrifugation at 1,400 x g for 10 minutes. TCA was added at 10% final concentration to the supernatants and the mixture was incubated at –20 oC for one hour to precipitate the extracellular proteins. The proteins were then separated by centrifugation at 15,000 x g for 20 minutes at 4 oC. Protein pellets were washed twice with ice-cold acetone and the residual acetone was removed by air-drying. Protein pellets were resuspended in 10 mM Tris-HCl (pH 7.5). For intracellular protein analysis, the cell pellets were harvested by centrifugation at 1400 x g for 10 minutes, washed in P buffer [32] and sonicated on ice for three bursts of 5 s and re-suspended in P buffer containing the EDTA-free protease inhibitor cocktail (Roche).

For Western blot analysis, proteins were fractionated by SDS-PAGE in 10% and 12% (w/v) acrylamide gel [33] and transferred onto immobilon polyvinylidene difluoride membranes (Millipore), as described [34]. The transferred material was incubated with rabbit polyclonal antibodies raised against *S*. *lividans* TK21 mature alpha-amylase with a molecular size of 59 kDa (AmlB; a gift from C. Isiegas) or *S*. *coelicolor* mature agarase with a molecular size of 32 kDa (DagA; [35]) followed by incubation with HRP-conjugated protein A (Invitrogen Laboratories) diluted 1:10,000 in PBS containing 5% (w/v) skimmed milk for 40 min at room temperature [36]. Peptides reacting with the antibodies were revealed using the ECL enhanced chemiluminescence system from Amersham after one min incubation and different exposures to X-ray film ranging from 20 s to 3 min.

### Enzyme activities

To determine extracellular alpha-amylase and agarase activity, the supernatants from the aliquots of bacterial cell cultures were concentrated at the indicated phases of growth by precipitation with ammonium sulphate brought to 80% saturation; the precipitated protein was collected by centrifugation at 13,000 x g for 30 min and dissolved in 20 mM phosphate buffer (pH 7.0) for alpha-amylase and in 50 mM imidazole-HCl (pH 6.5) for agarase. Alpha-amylase and agarase activities were determined as previously described [16, 35]. One unit of enzyme activity is the amount of enzyme that increased absorbance at 540 nm (alpha-amylase) or at 450 nm (agarase) by 0.001 per minutes of incubation under the assay conditions. The specific activity was expressed as units per mg of dry cell weight. The enzyme activities used for representations are the average values of three independent experiments.

## Supporting Information

S1 Fig Sporulation phenotype of the overproducer strains. Sporulation phenotype of the *S*. *lividans* TK21 (pIJ486), *S*. *lividans* TK21 (pAMI11) and S. *lividans* TK21 (pAGAs5) after 3 and 7 days’ growth in MS medium at 30°C (a) and in R5 medium (b).
(TIF)

S1 Table Oligonucleotide primers used for gene transcript amplification. To be identified more clearly, the *S*. *coelicolor* gene nomenclature has been adopted for *S*. *lividans*, and the SCO acronym has been changed to SLI to indicate the strain of origin.
(DOC)
