# Supplementary figures and images for: Overproduction of a Model Sec- and Tat-Dependent Secretory Protein Elicits Different Cellular Responses in Streptomyces lividans
Source: PLoS One. 2015 Jul 22;10(7):e0133645. doi: 10.1371/journal.pone.0133645 (PMC4511581; doi:10.1371/journal.pone.0133645)

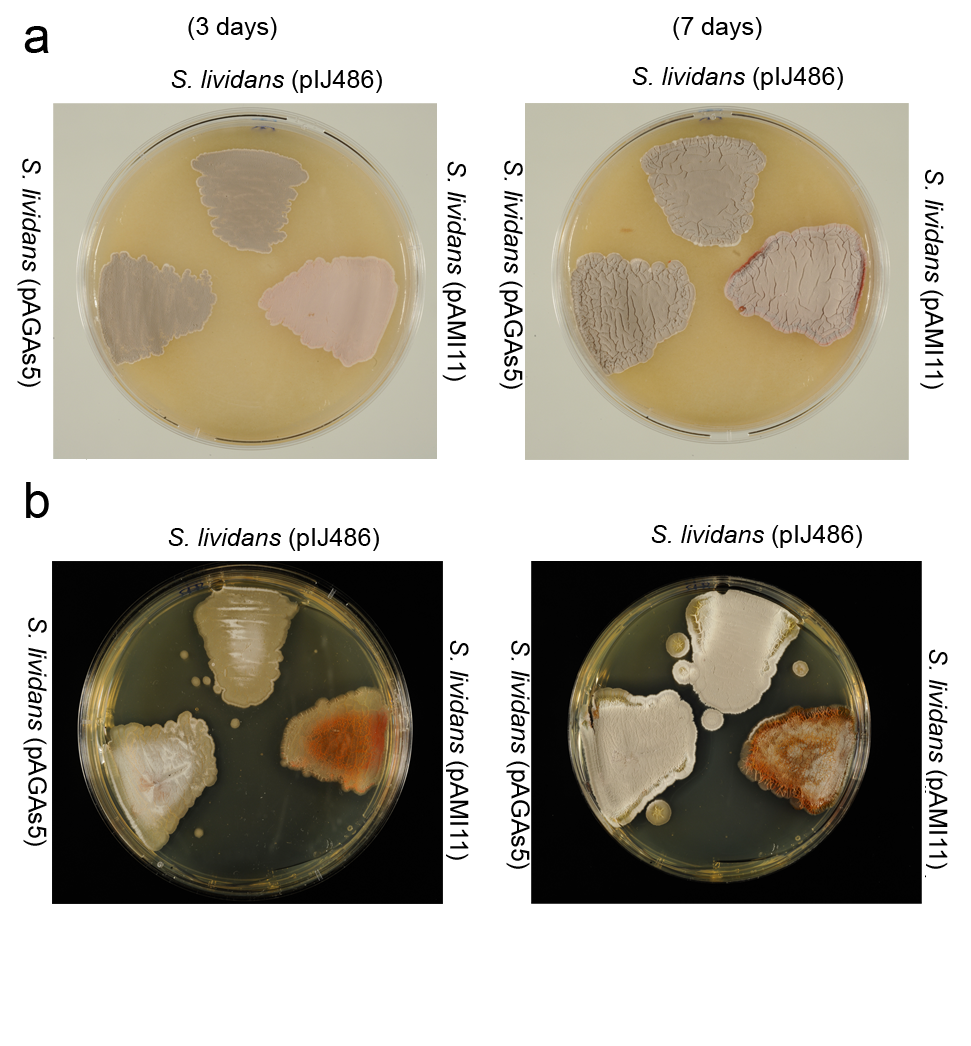

Supplement: S1 Fig — Sporulation phenotype of the S. lividans TK21 (pIJ486), S. lividans TK21 (pAMI11) and S. lividans TK21 (pAGAs5) after 3 and 7 days’ growth in MS medium at 30°C (a) and in R5 medium (b). (TIF) [file pone.0133645.s001.tif]
