# Supplementary material for: Overproduction of a Model Sec- and Tat-Dependent Secretory Protein Elicits Different Cellular Responses in Streptomyces lividans
Source: PLoS One. 2015 Jul 22;10(7):e0133645. doi: 10.1371/journal.pone.0133645 (PMC4511581; doi:10.1371/journal.pone.0133645)
Supplement: S1 Table — To be identified more clearly, the S. coelicolor gene nomenclature has been adopted for S. lividans, and the SCO acronym has been changed to SLI to indicate the strain of origin. (DOC) [file pone.0133645.s002.doc]

**Table S1. Oligonucleotide primers used for gene transcript amplification**

| **Gene*** | **Forward primer** | **Reverse primer** | **Reference** |
| --- | --- | --- | --- |
| SLI1513 (*relA*) | GCGAGAAGCAGGAGAAGAAG | CTCCAGCTCCCACTTGATG | [13] |
| SLI1632 (*tatC*) | CGAAGGTGCTGATCGAGTT | AAGGAGAGACCGAAGACGAC | This work |
| SLI2151 (*cox*) | TTCATCGTCACCTTCGTGAT | GTCGGACGAGAGCGAGAT | This work |
| SLI4721 (*rplO*) | GCTTCAAGAACCCGTTCAA | GACCTTGACGAGGCTGTTCT | This work |
| SLI4727 (*rpsM*) | CGAGTACGTCGACAACAACA | CCTGGTAGGTGCCGATCT | This work |
| SLI4808 (*sucC)* | ACACGGTCCACAAGGTGAT | TCGAGGAGGAAGGAGACGTA | This work |
| SLI5369 *(atpf*) | CCTGATCGCCTTCGTCAT | ACGCTCTTCCAGAACCTTGT | This work |
| SLI5776 (*gluB*) | CTGCAGAAGTACCCGACGTA | TTGAACTTGCCCTTGAACTG | This work |

To be identified more clearly, the *S. coelicolor* gene nomenclature has been adopted for *S. lividans*, and the SCO acronym has been changed to SLI to indicate the strain of origin.
